# Aged mice ovaries harbor stem cells and germ cell nests but fail to form follicles

**DOI:** 10.1186/s13048-022-00968-4

**Published:** 2022-03-23

**Authors:** Diksha Sharma, Deepa Bhartiya

**Affiliations:** Stem Cell Biology Department, ICMR- National Institute for Research in Reproductive Health, Jehangir Merwanji Street, Mumbai, 400 012 India

**Keywords:** Ovary, Stem cells, Germ cell nest, VSELs, OSCs, Ageing, Neo-oogenesis

## Abstract

**Background:**

We recently published evidence to suggest that two populations of stem cells including very small embryonic-like stem cells (VSELs) and ovarian stem cells (OSCs) in ovary surface epithelium (OSE) undergo proliferation/differentiation, germ cell nests (GCN) formation, meiosis and eventually differentiate into oocytes that assemble as primordial follicles on regular basis during estrus cycle. Despite presence of stem cells, follicles get exhausted with advancing age in mice and result in senescence equivalent to menopause in women. Stem cells in aged ovaries can differentiate into oocytes upon transplantation into young ovaries, however, it is still not well understood why follicles get depleted with advancing age despite the presence of stem cells. The aim of the present study was to study stem cells and GCN in aged ovaries.

**Methods:**

OSE cells from aged mice (> 18 months equivalent to > 55 years old women) were enzymatically separated and used to study stem cells. Viable (7-AAD negative) VSELs in the size range of 2–6 µm with a surface phenotype of Lin^−^CD45^−^Sca-1^+^ were enumerated by flow cytometry. Immuno-fluorescence and RT-PCR analysis were done to study stem/progenitor cells (OCT-4, MVH, SCP3) and transcripts specific for VSELs (*Oct-4A, Sox-2, Nanog*), primordial germ cells (*Stella*), germ cells (*Oct-4, Mvh*), early meiosis (*Mlh1, Scp1)* and ring canals (*Tex14)*.

**Results:**

Putative VSELs and OSCs were detected as darkly stained, spherical cells with high nucleo-cytoplasmic ratio along with germ cells nests (GCN) in Hematoxylin & Eosin stained OSE cells smears. Germ cells in GCN with distinct cytoplasmic continuity expressed OCT-4, MVH and SCP3. Transcripts specific for stem cells, early meiosis and ring canals were detected by RT-PCR studies.

**Conclusion:**

Rather than resulting as a consequence of accelerated loss of primordial follicle and their subsequent depletion, ovarian senescence/menopause occurs as a result of stem cells dysfunction. VSELs and OSCs exist along with increased numbers of GCNs arrested in pre-meiotic or early meiotic stage in aged ovaries and primordial follicle assembly is blocked possibly due to age-related changes in their microenvironment.

**Supplementary Information:**

The online version contains supplementary material available at 10.1186/s13048-022-00968-4.

## Introduction

Young couples in present times, tend to delay parenthood for successful career and thus more and more couples in their thirties plan parenthood. It becomes important for the females to maintain a healthy ovarian reserve. Eventual loss of ovarian reserve with advancing age leads to menopause associated with multiple organ dysfunction including osteoporosis, cardiovascular disease, cancer etc. Several mechanisms have been put forth to explain loss of ovarian reserve including genetic, mitochondria dysfunction, telomere length shortening, oxidative stress, stromal fibrosis etc. However, it is still not clear what exactly leads to ovarian aging.

In-depth understanding of the mechanisms driving ovarian aging is of critical importance. Towards this, a recent scRNA-seq study on young and aged cynomolgus monkey ovaries reported that ovarian aging mainly occurs due to oxidative stress [1]. 7 ovarian cell types were detected including oocytes, granulosa cells, stromal cell, smooth muscle cells, endothelial cells, NK cells, macrophages collected by centrifuging cells at 1000 rpm (200-250 g). Oxidative damage was identified as a crucial factor that results in ovarian functional decline with age. In addition, inactivated anti-oxidative pathways, increased reactive oxygen species, and apoptosis were observed in granulosa cells from aged women. Two key antioxidant genes (IDH1 and NDUFB10) showed reduced expression. However, Wang et al. [1] failed to delineate contributions (if any) of stem cells during ovarian aging. This is because, they subjected cells collected by spinning at 1000 rpm for scRNA-seq and we have discussed that stem cells can be collected only when cells are centrifuged at 3000 rpm (1000 g) [2–4].

Niikura et al. [5] showed presence of pre-meiotic germ cells in aged mice ovaries. Genes associated with meiotic commitment, *stimulated by retinoic acid gene 8* (*Stra8*) and *deleted in azoospermia‐like* (*Dazl*), were highly expressed in aged mouse ovaries. However, histological studies failed to show the presence of oocytes, suggesting that *Stra8* and *Dazl* positive pre-meiotic germ cells do not undergo further differentiation. These germ cells generated NOBOX positive oocytes and follicles when grafted into young adult wild‐type female mice ovaries. Stem cells have also been enriched from aged, menopausal human ovaries as DDX-4 positive cells by MACS [6]. These stem cells upon in vitro culture entered meiosis and differentiate into oocyte-like structures. Upon grafting in human ovarian cortex, GFP and DDX-4 positive stem cells differentiate into GFP positive oocyte-like structure in vivo.

Two populations of stem cells exist in adult ovaries including very small embryonic-like stem cells (VSELs) and ovarian stem cells (OSCs) in the ovary surface epithelium (OSE) [7]. We recently deciphered various events, well documented in fetal ovaries, by studying the stem cells located in the OSE of adult mice ovaries [2]. Present study was undertaken to study the presence of VSELs, OSCs and GCN in aged ovaries.

## Study design and methods

Aged, female Swiss (> 18 months old) mice, maintained in the Institute Experimental Animal Facility were used for the present study, after obtaining permission from Institute Animal Ethics Committee. The animals were housed under controlled temperature of (23 ± 1^◦^C) and humidity (55 ± 5%), with 14 h light/10 h dark cycle with free access to chow and water. Aged mice ovaries were used to study the presence of stem cells including VSELs, OSCs and germ cell nests in the ovary surface epithelium as reported recently in normal adult ovaries [2].

### Histological studies

The ovaries were fixed in neutral buffer formalin (NBF) for 48 h at room temperature, washed with PBS, passed through ascending series of methanol and later paraffin blocks were prepared for histological studies using standard methods. 5 μm thick sections were cut and stained with Hematoxylin and Eosin (H&E) for viewing under a bright-field microscope (90i NIKON). Representative fields were photographed using Nikon 90i microscope and data was recorded. In few mice, ovaries were bigger in size and appeared cystic with cancer-like changes.

### OSE cells smear preparation

Intact ovaries were placed singly in 500 µl of 0.25% trypsin EDTA (Gibco) for 30 min at 37 °C. Different methods were used to separate the OSE cells from the intact ovaries and direct trypsinization provided best results [2]. The reaction was stopped with equal volume of DPBS supplemented with 20% FBS (Gibco). The cell suspension was pipetted up and down, vortexed for few seconds and the denuded ovaries were removed after 5 min [2] The cells suspension was spun at 250 g for 15 min followed by spinning the supernatant at 1000 g for 15 min. Later, pellet obtained by spinning at both the speeds were mixed together, passed through 40 μm filter and used for preparing smears on poly-lysine coated slides. For this, cells were placed in a small pre-marker area and after air drying, were fixed in 4% PFA (Sigma) for 15 min, washed three times with PBS, air dried and stored at 4 °C for H&E staining and immunofluorescence studies. Reason for collecting cells at two different speeds of centrifugation, is because the bigger somatic cells (with abundant cytoplasm) and germ cell nests are first collected by spinning at 250 g since these cells may get damaged and burst when spun at higher speed. Later the supernatant is centrifuged at 1000 g to collect smaller sized VSELs with minimal cytoplasm and germ cell nests which can otherwise be lost [3]. Mixing cells collected at two different speeds enabled all the cells in the OSE to be visualized in the smears. These cells were also used to extract RNA.

### VSELs enumeration by flow cytometry

Briefly, ovaries were cleared of surrounding fat, rinsed with DPBS and processed to obtain single-cell suspension. This protocol was recently described and used to study stem cells across estrus cycle in adult ovaries [2]. In brief, ovaries were minced and kept at 37 °C for 20 min in 1 mg/ml type IV collagenase solution (Gibco). After adding DPBS containing 20% FBS (Gibco), the cells suspension was filtered through 40 μm filter and cells were collected by centrifuging at 250 g and 1000 g as described above. RBCs lysis was carried out if cell pellet appears to be red in color. RBC lysis solution (1X) was added to the cell pellet for 10 min in dark followed by dilution with D-PBS and cells were collected by centrifugation at higher speed. Cells pellet was then re-suspended in DPBS containing 2%FBS and then incubated with a cocktail of antibodies including FITC-conjugated rat anti mouse Sca-1 (#553335, BD Biosciences), PE-conjugated rat anti mouse CD45 (#553081, BD Biosciences), APC-conjugated mouse lineage antibody cocktail (#558074, BD Pharmingen) for 20 min followed by washing in DPBS. Cells were then transferred to round bottom tubes for flow cytometric analysis. Unstained tube was prepared by omitting primary antibody. VSELs in the aged ovaries were enumerated by flow cytometry as 7-AAD negative, 2–6 µm cells with a surface phenotype of Lin^−^CD45^−^Sca-1^+^. Approximately one hundred thousand cells were subjected to flow cytometry analysis. Calibration size beads (Invitrogen) were used to gate 2–6 µm events and unstained sample was run to check for auto-fluorescence. FMO controls were run to determine the cutoff point between background fluorescence and positive fluorescence for FITC. The samples were run on FACS Aria and results were analyzed using BD FACS Diva software. The experiment was repeated on three different samples and always in technical duplicates.

### Immunolocalization of stem/germ cell markers

Immunofluorescence studies were carried out to study the expression of OCT-4 (1:100, Santa Cruz, sc-5279), MVH (1:400, Abcam, ab13840) and SCP-3 (1:400, Abcam, ab15093) in OSE cell smears. Smears were first rinsed thrice with TRIS buffered saline (TBS) for 5 min each. Permeabilization with 0.2% of triton X- 100 was carried out for 20 min only for SCP-3 staining. Cells were then blocked with 3% BSA for 1 h at room temperature in a humidified chamber and incubated with primary antibody diluted in Antibody Signal Enhancer Solution at 4 °C in a moist chamber for 12–16 h as described earlier [8]. Antibody Signal Enhancer was prepared by mixing 10 mM Glycine, 0.1% Tween 20, 0.1% H_2_O_2_ (3% stock solution) in 1 ml of TBS. The primary antibody was omitted from the negative controls. Next day, the cell smears were brought to room temperature, washed with wash buffer [0.1% tween-20 in TRIS buffer saline (TBS)] three times for 5 min each. This was followed by incubation with secondary antibody (Alexafluor568; 1:500, diluted in 0.1% of Tween-20) for 2 h at room temperature in dark. The cells were washed five times with TBS-Tween 20 for 5 min each and counterstained with DAPI (Thermo Fisher) for 25 min. Finally, cells were rinsed thrice with TBS for 5 min each, mounted with Vectashield (Vector labs) and images were captured by laser scanning confocal microscope (Olympus).

Immuno-localization studies were also done on paraffin sections. Sections were first cleared of wax by keeping the slides in xylene followed by rehydration in graded ethanol series. Antigen retrieval was performed in citrate buffer (pH 6) in a microwave. To quench autofluorescence, sections were treated with 0.1 M glycine for 1 h. This was followed by 3 washes with TBS (5 min each), blocking, primary antibody incubation, washing, secondary antibody (Alexafluor-568;1:500) and counterstaining with DAPI as described above. Primary antibody used included OCT-4. Primary antibody was omitted in negative control.

### RT-PCR studies to detect stem/germ cells specific transcripts

Markers specific for VSELs (*Oct-4A, Sox-2, Nanog*), primordial germ cell (*Stella*), OSCs (*Oct4, Mvh*), prophase of meiosis 1 (*Mlh-1, Scp-1*) and ring canals (*Tex-14*) in germ cell nests were studied by RT-PCR. Aged ovaries were placed in Trizol (Invitrogen, Carlsbad, CA, USA) at -80°C for RNA isolation using standard protocol. 500 ng of RNA pre-treated with DNase I (Fermentas) was used to prepare cDNA using the iScript cDNA synthesis Kit (Bio-Rad, USA, Hercules, CA, USA) according to the manufacturer’s directions. RNA was also extracted from OSE cells which were isolated as described above.

RT-PCR was carried out on CFX96 PCR machine using SYBR green chemistry (Bio-Rad) and 18S was used as housekeeping gene. PCR conditions were 1 cycle at 94 °C for 3 min followed by 40 cycles, with each cycle consisting of denaturation at 94 °C for 10 s, annealing at the specified temperature for 20 s, and extension at 72 °C for 30 s followed by melt curve analysis. The PCR products were later examined on a 2% agarose gel. The product size was estimated using a 100 bp DNA ladder (Bangalore Genei). The negative control did not include cDNA in the reaction mixture. Primer details and annealing temperatures for various transcripts are provided in the Table 1.

**Table 1:** Details of primers used in the study

Gene name	Sequence	Annealing temp °C	Product Size
*Oct-4*	F: CCTGGGCGTTCTCTTTGGAAAGGTG R: GCCTGCACCAGGGTCTCCGA	61	177
*Oct-4A*	F: AACCGTCCCTAGGTGAGCCG R: CCCACCTGGAGGCCCTTGGAA	62	190
*Sox2*	F: AGGAGTTGTCAAGGCAGAGAAGAGA R: GCCGCCGCGATTGTTGTGATT	62	167
*Nanog*	F: CAGGAGTTTGAGGGTAGCTC R: CGGTTCATCATGGTACAGTC	61	223
*Mvh*	F: AGTGGAAGTGGTCGAGGTGGT R: TGCCGGTGGTGCATCATGTCC	61	249
*Stella*	F: ACGCTTTGGATGATACAGACGTCC R: GCGCTTTGAACTTCCCTCCGGA	62	175
*18 s*	F: GGAGAGGGAGCCTGAGAAAC R: CCTCCAATGGATCCTCGTTA	61	177
*Mlh1*	F: TTGCCAACCTGCCAGATCTA R: ATTTGCAGCCAATCCACAGG	60	231
*Scp1*	F: GACAACGGCCCAGGAGGCA R TCTGCGGTTTCACGGCGGA	57	100
*Tex 14*	F: ACTAAGTGCCTGTGGCTGGAAG R: GAGTGGATGGTAACTGCTCACC	61	137

## Results

### Morphological changes in aged ovary

**Fig. 1:**
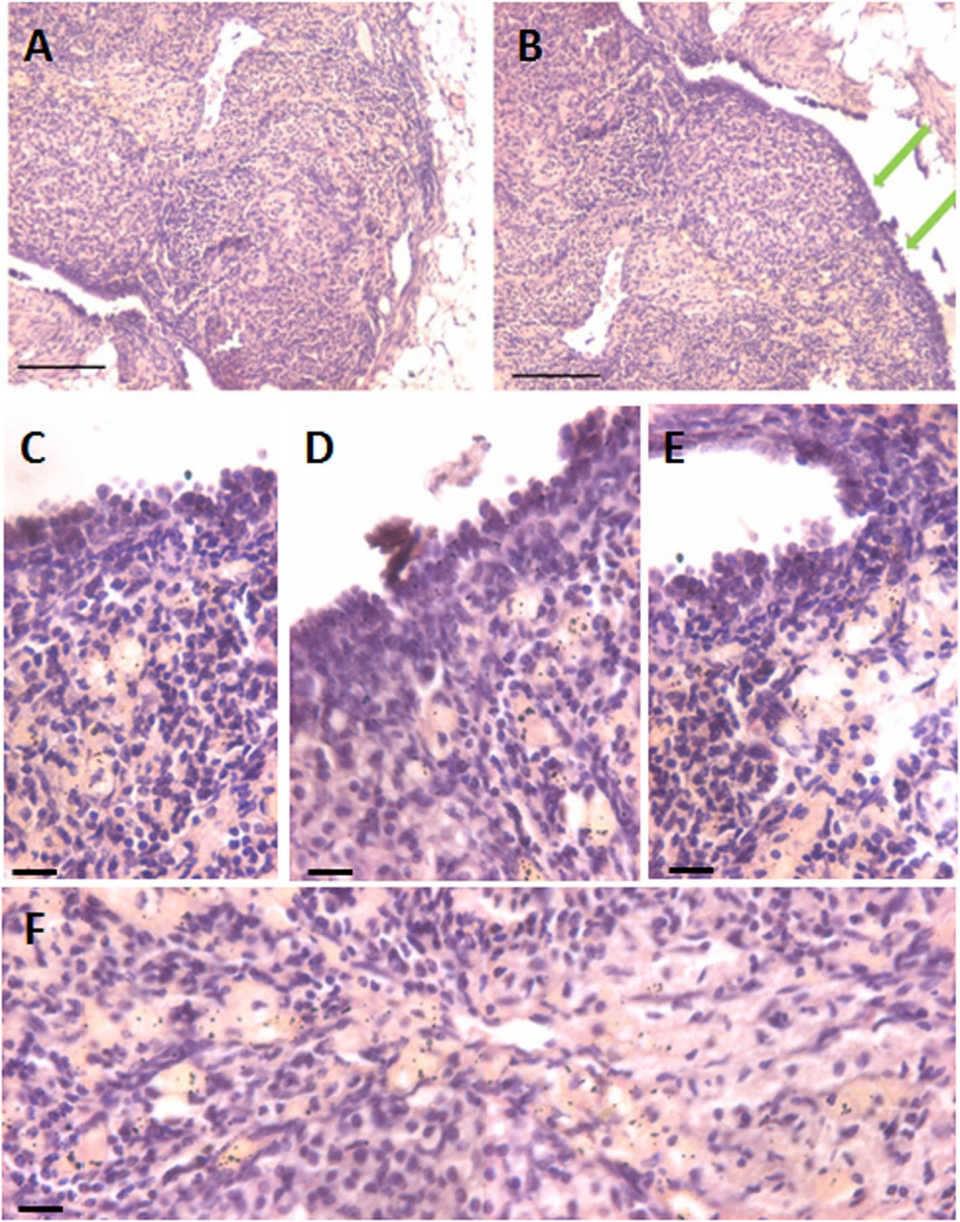
**H&E** stained sections of > 20 months aged mice ovaries. **A**. The ovary comprised prominently of stroma and lacked follicles and corpora lutea. **B**. The OSE cells were prominently observed (green arrows) **C-E**. Different fields of ovarian sections showing prominent OSE layer. **F.** Stromal compartment was highly vascularized. Scale: 20 µm

**Fig. 2:**
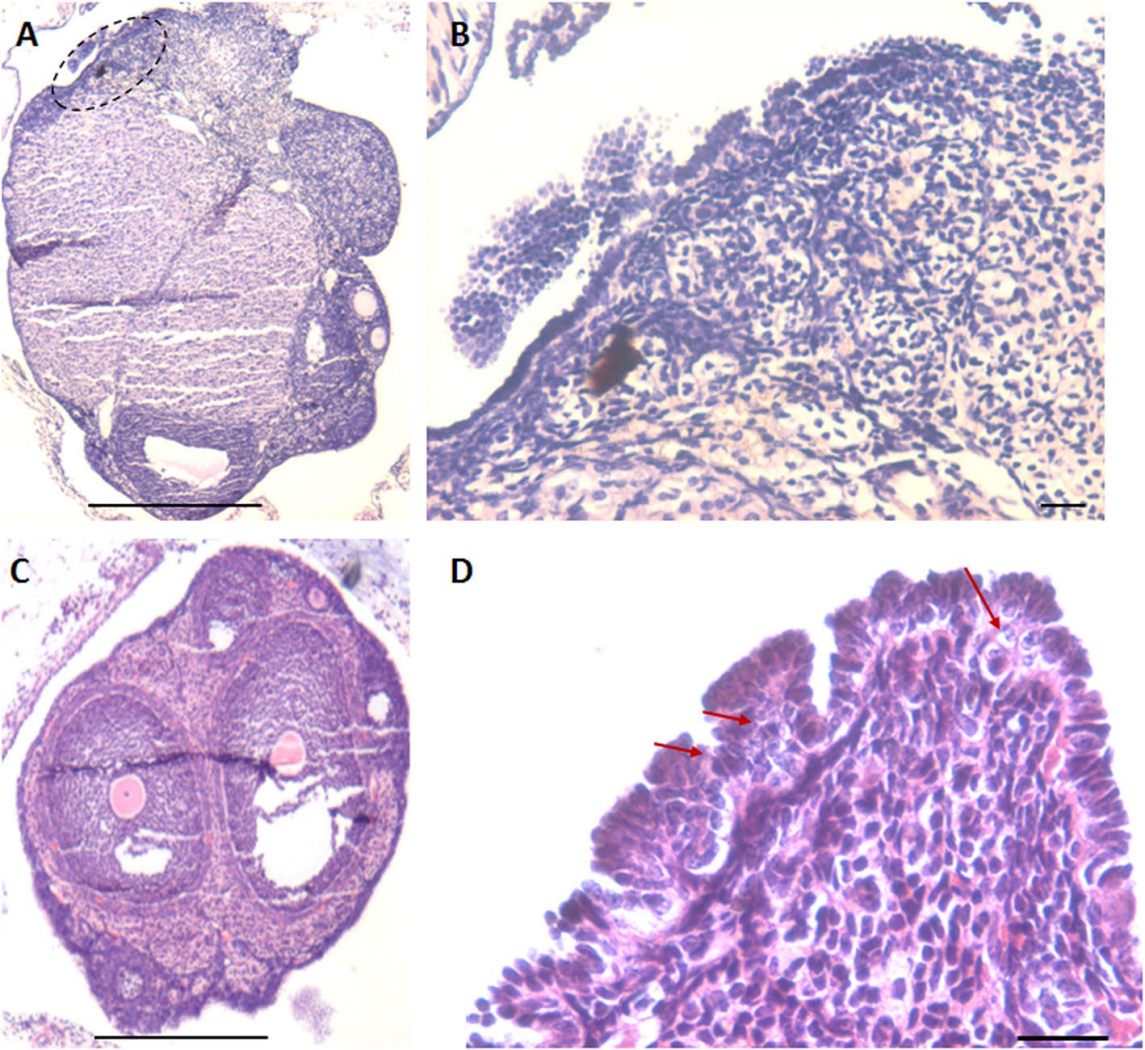
Aged ovaries showing hyperplasia of OSE. **A-B.** 18 months old, aged mouse ovary with excessive hyperplasia of the OSE. Few follicles were also observed. **C**. Six months old mice ovary with follicles in different stages of growth and maturation **D.** OSE cells in 20 months old ovary showing distinct hyperplasia and epithelial cells were interspersed by a distinct population of putative germ cells (red arrow). Scale: 20 µm

**Fig. 3:**
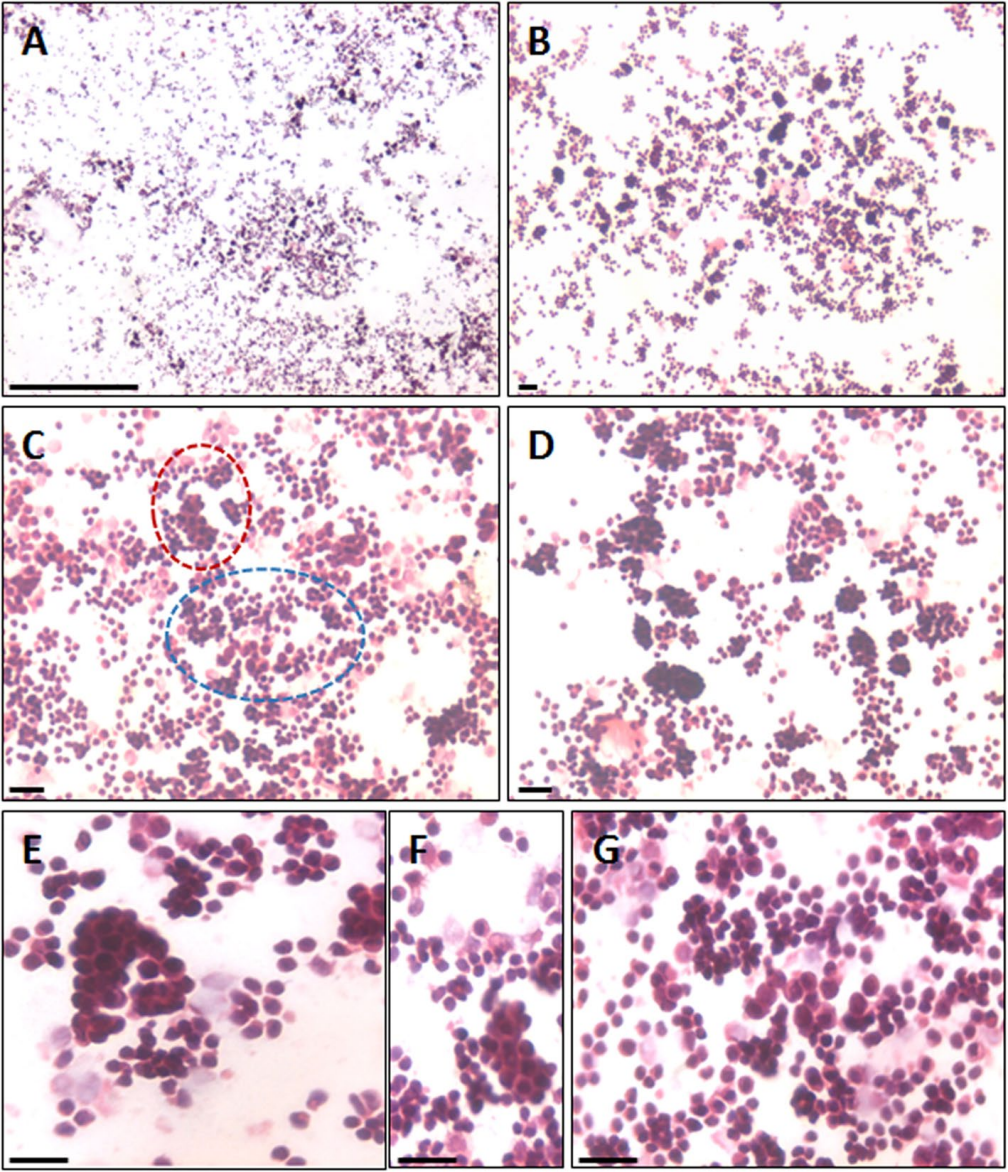
Representative H&E images of OSE cells smears. **A-G**. Large numbers of cells were observed singly and as small clusters with cytoplasmic continuity. These clusters appear to be the putative germ cell nests. Stem cells and germ cell nests were darkly stained with H&E. OSE cells smears were prepared from 24 months old mice ovaries. **F **and** G** are the higher magnification images of GCN encircled in red and blue respectively in **C**. The experiments were repeated three times. Scale: 20 µm

**Fig. 4:**
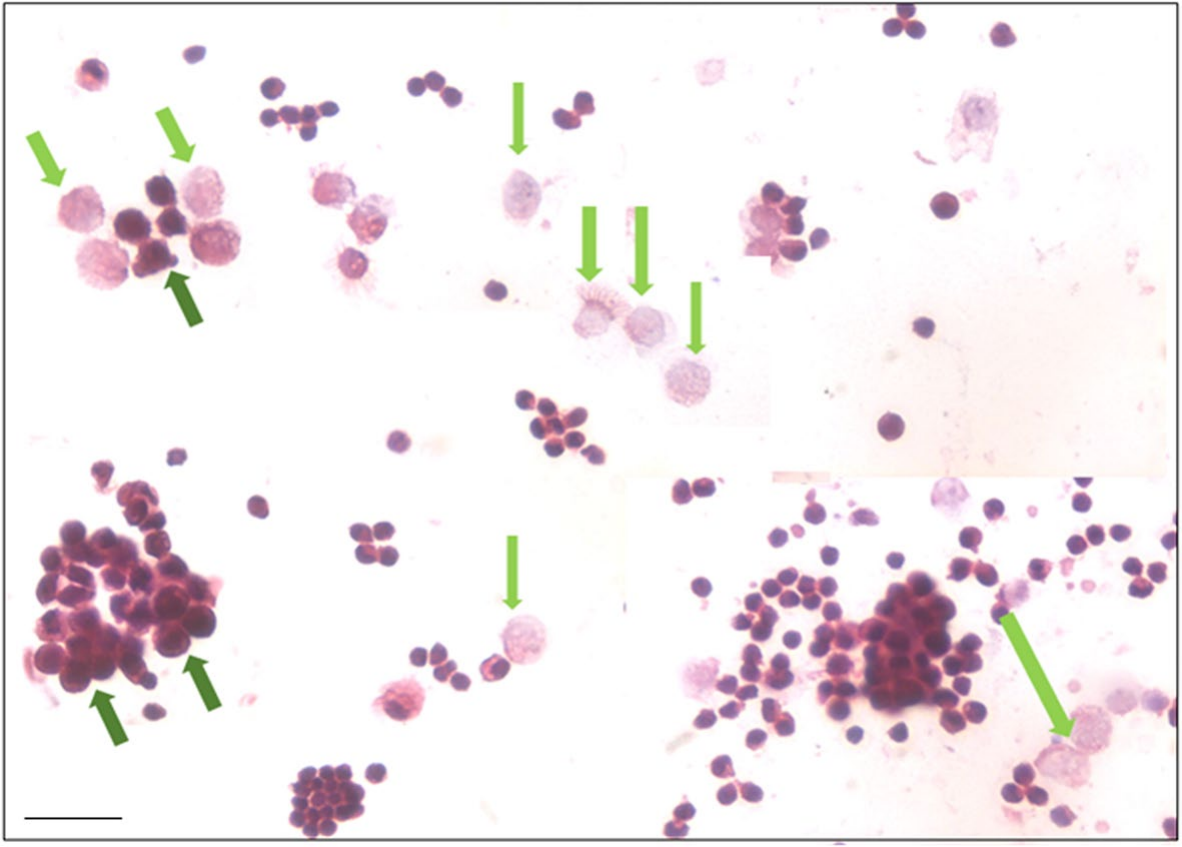
H&E stained OSE cells smears show the presence of stem cells, germ cell nests (GCN, dark green arrows) and putative unenclosed oocytes (light green arrows). At higher magnification, the cytoplasmic connectivity amongst the cells comprising the putative GCN was clearly visualized. The GCN were of varying sizes and were apparently in different stages of development. The experiment was repeated three times. Scale: 20 µm

**Fig. 5:**
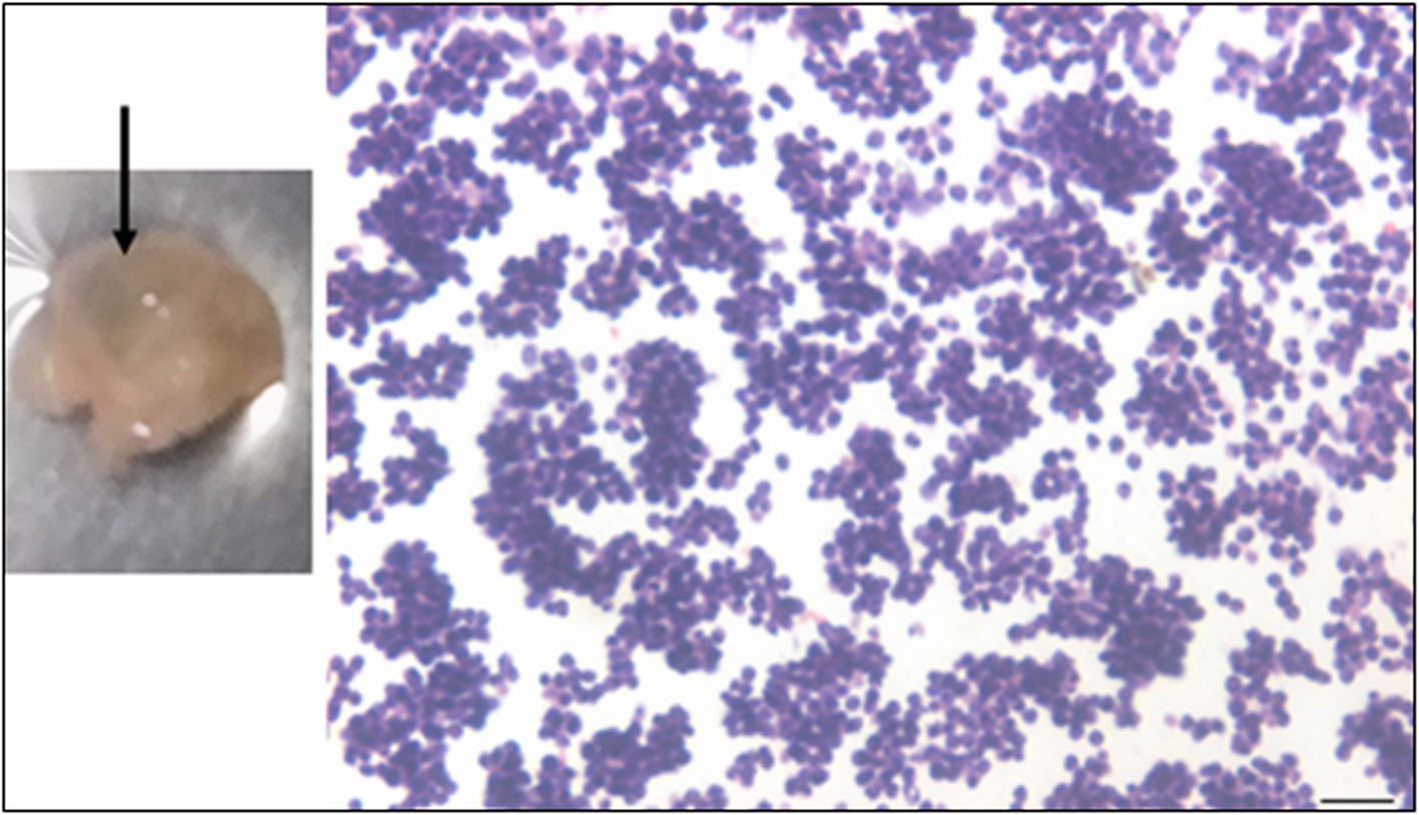
Cystic fluid smear from an aged (20 months old) ovary. Large numbers of putative stem cells were observed (mostly as clusters) in cells smears prepared from the cystic fluid after H&E staining. Scale: 20X

H&E stained ovarian sections of aged ovaries (> 20 months) completely lacked follicles (Fig. 1A) including primordial follicles in the cortical region and even corpus lutea were not observed. There was extensive, highly vascular stroma with fibrosis at places. The OSE cells showed hyperplasia and appeared multilayered (Fig. 1B-E). At higher magnifications, small, spherical, darkly stained cells with minimal cytoplasm were observed (Fig. 1C). These could be the putative stem cell with high nucleo-cytoplasmic ratio and of variable size since they were cut in different plane of sections. Stroma appeared to be highly vascularized (Fig. 1F). Fig. 2 shows prominent OSE of another aged ovary, comprising of a prominent layer of columnar epithelial cells lining the ovarian surface (Fig. 2A-B). Stromal fibrosis was clearly evident with very few follicles. In comparison to aged mice, six months old mice ovary has large numbers of follicles in different stages of maturation and minimal stroma (Fig. 2C). A careful viewing of OSE cells showed it was interspersed by cells of distinct morphology (Fig. 2D). Further studies were done to study cells present in the OSE by making cells smears. Large numbers of cells were isolated from the ovary surface as shown in Fig. 3. Darkly stained spherical cells were the putative stem cells and pale blue stained cells with pale pink cytoplasm were the epithelial cells. Stem cells were present singly and as clumps which may be the putative germ cell nests (GCN). Large numbers of GCN were observed scattered throughout and were of different sizes. At higher magnifications, epithelial cells were clearly visualized along with large numbers of dark stained cells which are the stem cells in different stages of differentiation. These possibly represent growth arrested pre-meiotic germ cell nest or early meiotic nests. At higher magnification (Fig. 4), the cytoplasmic connectivity amongst the cells comprising the GCN was clearly visualized. The cells comprising the GCN were of varying sizes and were apparently in different stages of development. Besides, several bigger cells were visualized which could be the unenclosed oocytes. In few mice, the ovaries were enlarged and appeared cancerous in accordance with published literature that risk of developing ovarian cancer increases with age [9]. The ovaries appeared cystic and the cystic fluid was collected with a syringe and used to prepare cell smear which was stained with H&E stained using similar protocol as described above for OSE cells. The cystic fluid had a large number of putative stem cells, mostly present as clusters (Fig. 5). Similar cells have been reported in human ovarian cancer samples [10] but further studies need to to be undertaken to characterize these cells in details.

### Enumeration of VSELs by flow cytometry

**Fig. 6:**
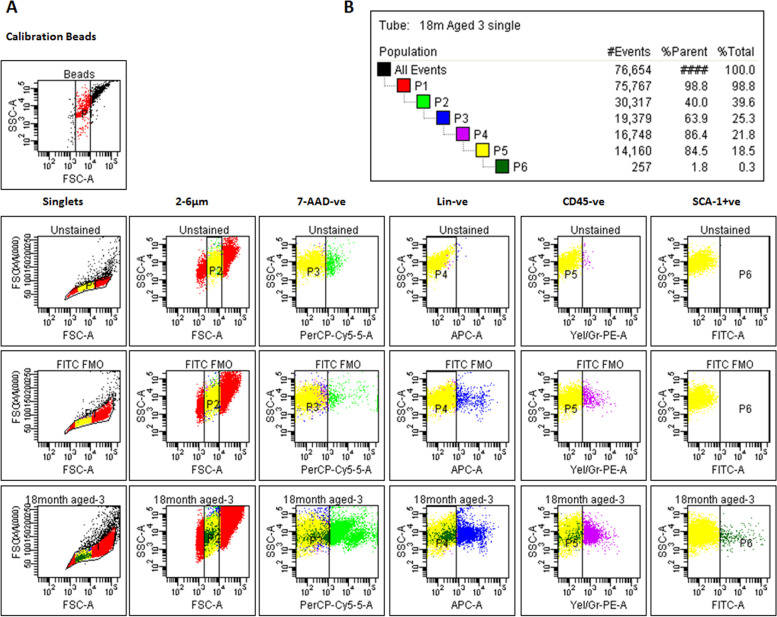
Enumeration of VSELs in 18 months old aged mice ovaries. VSELs were studied in 18 months old aged ovary as cells in the size range of 2–6 µm with surface phenotype of Lin^−^CD45^−^Sca-1^+^. Unstained controls were run to determine negative population and FITC FMO controls were run to identify positive population. Results of three different experiments show that 0.33 ± 0.08% (Mean ± SE) of total cells in ovary were VSELs

Intact ovaries were processed and used to enumerate VSELs by flow cytometry and 0.33 ± 0.08% of total cells were the VSELs (Fig. 6). Earlier we have reported that VSELs show distinct change in numbers across estrus cycle in the mice ovaries [2] as well as in the uterus [11]. Percent events reported across estrus cycle in the adult ovaries were 0.16 ± 0.03% during proestrus, 0.41 ± 0.05% during estrus, 0.96 ± 0.03% during metestrus and 0.36 ± 0.06% during diestrus [2] whereas the aged mice did not show cyclicity.

### Immunoexpression of stem/germ cell markers

**Fig. 7:**
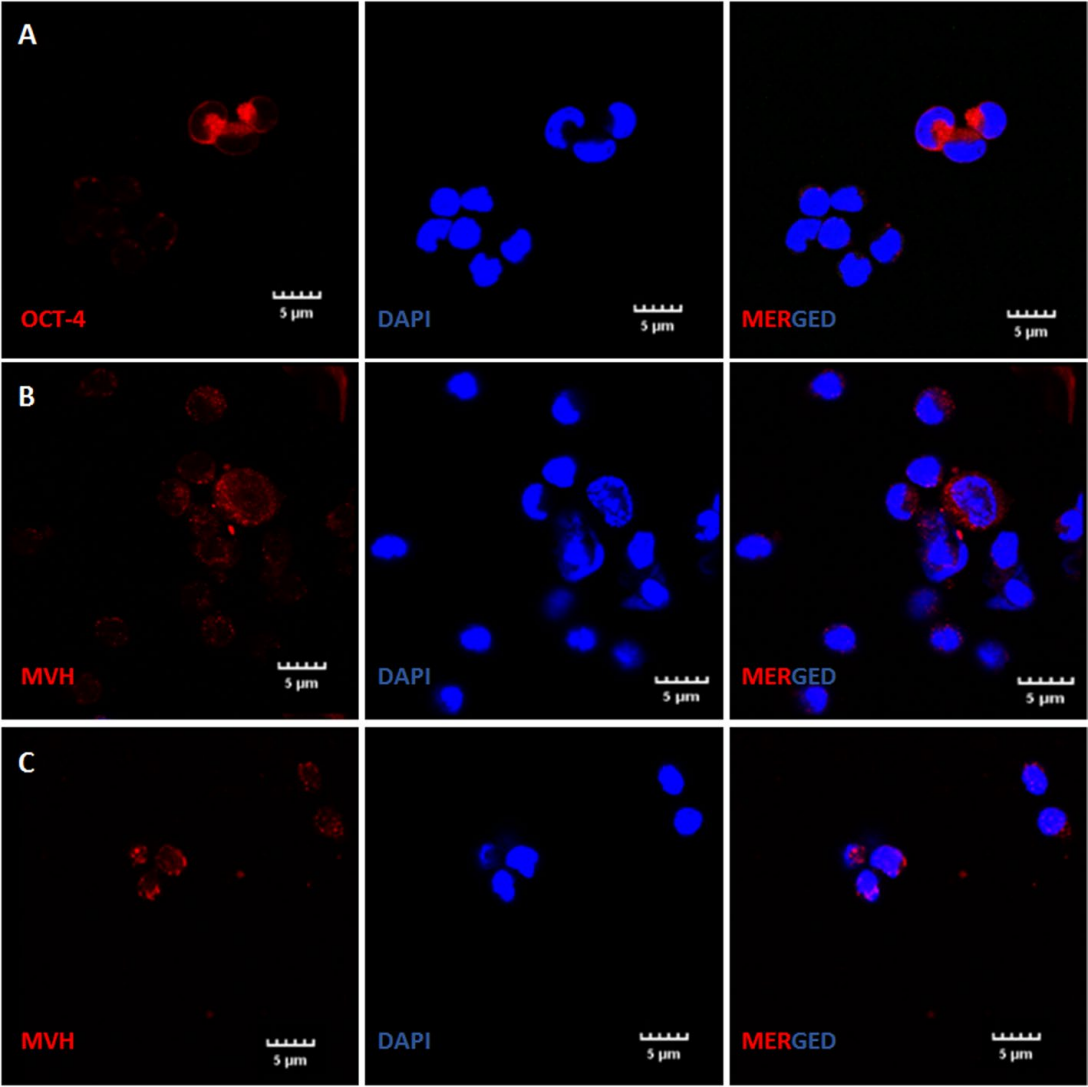
Immuno-expression of OCT-4 and MVH. Confocal microscopy showed that germ cell nests persist in 24 months old aged ovary. **A.** OCT-4 positive germ cell nest **B-C**. MVH positive germ cells in aged OSE smear. Scale: 5µm

**Fig. 8:**
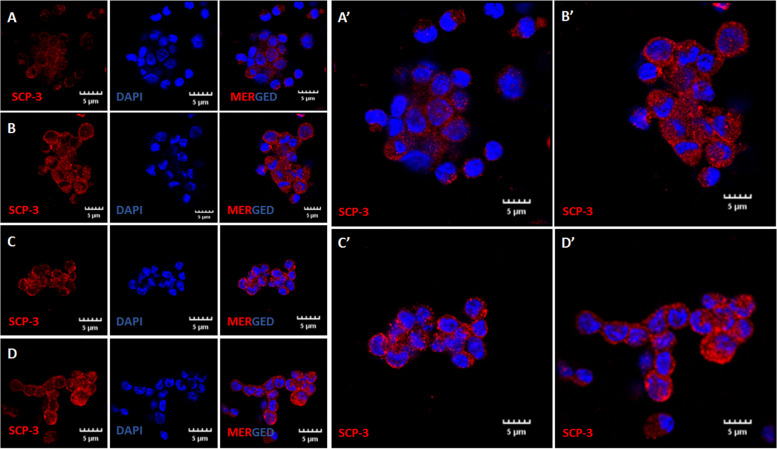
Immuno-expression of SCP-3. **A-D.** Meiotic germ cell nest of 24 months old mice as shown by SCP-3 staining. Note one of the oocytes in the nest grows bigger in size and will become the future egg. **A’-D’** are enlarged images of **A**-**D** to appreciate presence of cells of different sizes with cytoplasmic continuity. Scale: 5µm

**Fig. 9:**
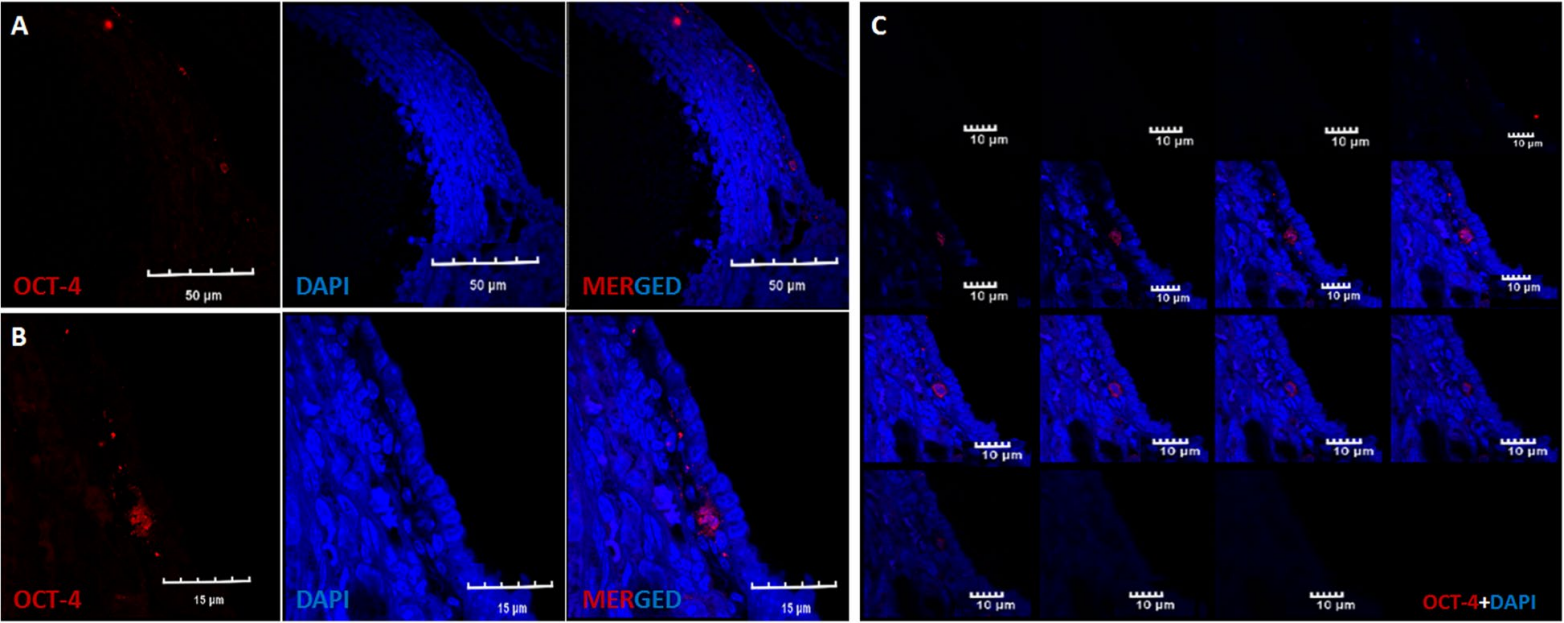
Immuno-expression of OCT-4 and MVH. **A-C.** Oct-4 positive stem cells and germ cell nest in 18 months old mice ovary sections. **A**. OCT-4 positive stem cells just beneath the OSE in aged mice. Scale: 50µm **B**. OCT-4 positive Germ Cell Nest. Scale: 15µm **C**. Z-stack of A. Scale: 10µm

OCT-4 and MVH expression was clearly visualized in clusters and germ cells respectively (Fig. 7A-C). Immunostaining with SCP-3 (Fig. 8A-D) indicated that these nests are arrested in prophase I of early meiosis. At places, single cells were bigger in size compared to the rest and were the putative developing oocytes. OCT-4 positive cells and germ cell nests were visualized just beneath the OSE in Fig. 9A-C. These results suggest that aged ovaries possess stem/progenitor cells and germ cell nests that are unable to assemble as primordial follicles.

### Detection of stem/germ cells specific transcripts by RT-PCR

**Fig. 10:**
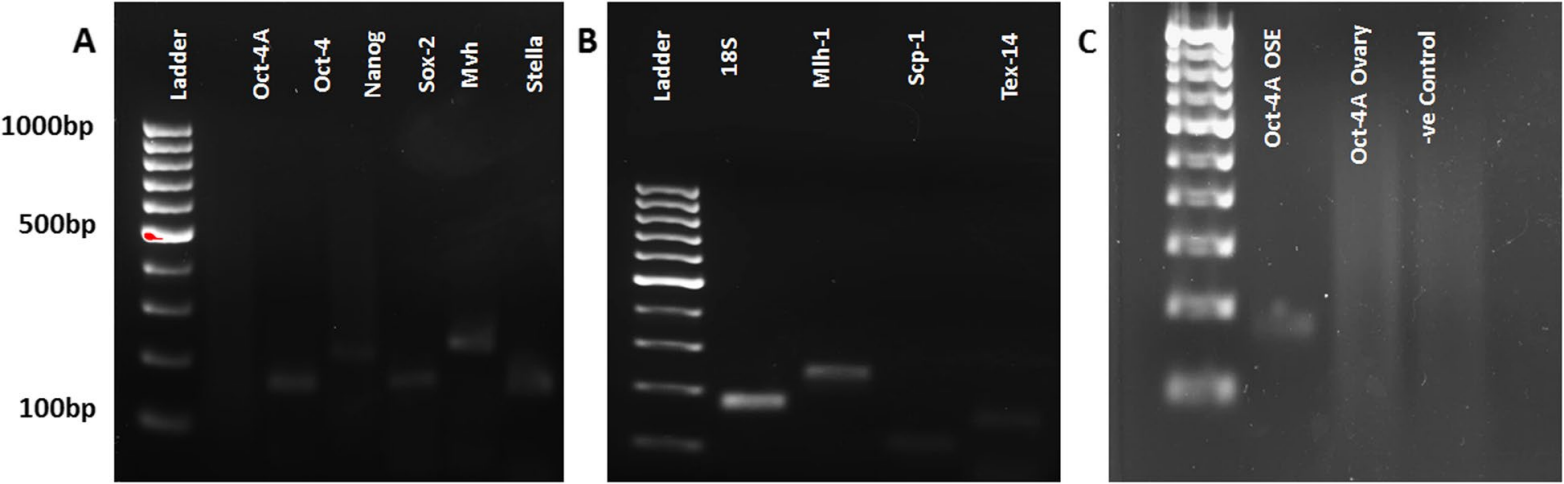
**A.** RT-PCR results for stem/germ cell markers. Transcripts specific for pluripotent stem cells *(Oct-4, Sox-2, Nanog*), germ cells *(Mvh)* and primordial germ cells *(Stella)* were detected in RNA extracted from intact 20 months old ovaries **B**. Detection of early meiotic markers (*Mlh1, Scp-1*) and marker specific for ring canals *(Tex14)* is suggestive of ongoing oogenesis. **C.** Faint expression of Oct-4A detected in RNA extracted from OSE cells but not in whole ovary. Negative control was run to check for genomic DNA contamination

RT- PCR studies were undertaken on 20 months old ovaries to study the stem cells and meiotic markers. Fig. 10 shows that transcripts specific for *Oct-4A*, *Oct-4, Sox-2, Nanog, Stella* and *Mvh* in RNA extracted from intact aged ovaries. *Oct-4A* (specific marker for pluripotent VSELs) was not detected possibly because of its minimal expression in intact ovary. However, its expression was detected in OSE, possibly due to the enrichment of the stem cells (Fig. 10C). Besides, expression of transcripts specific for *Scp-1, Mlh1* and *Tex14* along with *18 s* were also detected in aged ovaries (Fig. 10). *Scp-1* is the central element of synaptonemal complex and is known to be expressed in fetal ovary during early meiotic prophase 1. *Mlh1* is a marker for meiotic recombination and *Tex-14* is a marker specific for ring canals. Expression of all these markers indicates that nests in early meiotic stages do persist in aged ovary similar to adult ovaries [2].

## Discussion

In the present study we show that stem/progenitor cells including VSELs and OSCs exist and are functional in aged mice ovaries. Although the ovaries were devoid of follicles, the OSE remained prominent and expression of both OCT-4 and MVH was noted in the OSE cell smears. Similar small-sized stem cells have also been reported in post-menopausal human ovaries [12]. A large number of OCT-4 positive oogonial cells and SCP3 positive germ cell nests were observed and were apparently arrested in early-meiotic state. Transcripts specific for stem cells and early prophase I of meiosis and ring canals were detected. This indicated that germ cell nests persist in aged ovary but are unable to assemble as follicles. This eventually leads to senescence in mice and menopause in humans. These results could explain recent findings of Castillo-Fernandez et al. [13] who reported a distinct population of oocytes from aged mice with a ‘young-like’ transcriptome by studying single-cell transcriptome and methylome profile of young and aged mice ovaries. Young-like transcriptome is suggestive of early stages of differentiation of oocytes from the stem cells.

We have already published evidence that ovarian stem cells form primordial follicles on regular basis in young ovaries [2], and present study further shows that these stem cells are unable to function normally in aged ovaries, due to age-related niche changes. Depletion of follicles in aged ovaries is due to impaired differentiation of oocytes from the stem cells rather than the accelerated loss of oocytes which is the currently held view [14, 15]. Stem cells function normally but possibly their niche is affected which results in the blocked differentiation of oocytes. This was suggested in earlier reports also that stem cells when grafted in young ovarian cortex resulted in the formation of follicles in both mice [5] and humans [6]. Thus, ovarian aging indeed has a stem cell basis and it is the dysfunction and affected differentiation of stem cells due to a compromised niche that results in the loss of follicles in the aging ovaries. Our results are in addition to recent findings obtained by performing scRNA-seq on non-human primates which suggested that ovarian aging is mainly due to oxidative stress [1]. There is continuous replenishment of follicles in adult ovaries from the stem cells [2] and it is crucial to think of various ovarian pathologies in context of stem cells. The results are majorly based on morphology and provide scope for further research. Given that the rate of atresia is so high in the ovaries, post-natal oogenesis is necessary to compensate for the loss of follicles. We detected many growth-arrested oocytes in germ cell nests of 24 months old mice ovaries which otherwise lack primordial follicles. The impaired oocytes in the nests are unable to further differentiate into healthy oocytes and possibly undergo apoptosis/atresia resulting in follicle depletion. Regular atresia of follicles occurs in adult ovaries but neo-oogenesis from stem cells helps maintain follicle population in the young ovaries. Whereas in aged ovaries existing follicles undergo atresia and stem cells are unable to replace them and this possible results in menopause/senescence.

Niikura et al. [5] reported presence of pre-meiotic marker *Stra8* and *Dazl* expression in meiosis-committed cells, in aged mice ovaries which were otherwise depleted of oocytes and follicles. Grafting aged ovarian tissue in a young host resulted in the formation of NOBOX expressing follicles. Conversely exposure to the aged environment resulted in a decreased number of immature follicles in the young tissue. Stem cells were also enriched from aged, menopausal human ovaries as DDX-4 positive cells by MACS [6]. These stem cells upon in vitro culture entered meiosis and differentiated into oocyte-like structures. When grafted in human ovarian cortex, GFP + DDX-4 positive stem cells differentiated into GFP positive oocyte-like structure in vivo. Massasa et al. [16] discussed that stem cell niche failure rather than loss of oocyte stem cells is thought to be the cause of ovarian ageing.

Ten months old mice ovaries harbor many small follicles, exhibit extensive stromal fibrosis and remain in extended estrus in the presence of increased LH [17]. Stromal fibrosis has been reported in both mice and human ovaries with advancing age [18]. Stromal fibrosis, determined by picrosirius red staining for collagen I and III, along with up-regulation of inflammation associated transcripts was reported in aged mice ovaries and this alters the ovarian microenvironment [18]. This could be the underlying reason why ovarian stem cells present in the aged ovary are unable to differentiate normally and get arrested in early-meiotic state. Age-associated changes in the ovarian collagen and hyaluronan matrices observed in the mice ovaries also occur in human ovarian tissue [19].

A recent study has reported beneficial effects of transplanting MSCs in the aged rat ovaries [20]. Transplanting human placental derived MSCs to 52 months old rats could reverse ovarian aging. Multiple transplantation of MSCs was more effective and promoted primordial follicle activation and ovarian hormone (E2 and AMH) production than a single injection. There was a simultaneous increase in primordial follicle development and the number of primary and antral follicles. Tian et al. [21] reported improved ovarian reserve, follicle and blood vessel regeneration, reduced stromal fibrosis and normalized levels of sex hormones when MSCs from juvenile macaques were transplanted into elderly macaques. We recently discussed that upon transplantation, mesenchymal stromal cells (MSCs) help improve the niche by means of providing a paracrine support to the tissue-resident stem/progenitor cells including VSELs and OSCs in the ovaries [22]. Thus, MSCs improved the aged niche and the ovarian stem cells function normally as recently deciphered in adult ovaries by us [2]. Similarly, attempts have been made to treat aged mice ovaries with resveratrol, an anti-aging molecule [23, 24]. In comparison to age-matched control which produced no pups, young mice fed with resveratrol for 12 months were found to retain capacity to reproduce. They had greater number of follicles than controls. In addition, telomerase activity, telomere length and age-related gene expression in ovaries of mice fed with resveratrol resembled those of young mice. Resveratrol also helped improve the number and quality of oocytes [25]. Similar findings for resveratrol have been reported by other groups as well [26, 27]. Thus, two interesting options to improve ovarian reserve in aged ovaries include transplantation of mesenchymal stromal cells and by treating with resveratrol. Detailed studies are warranted to investigate effects of these strategies on VSELs and OSCs.

Ovarian cancers are the 5^th^ leading cause of cancer associated deaths in women and the most lethal of all the gynecological malignancies. Incidence of ovarian cancer is highest in peri- and postmenopausal women and more than 95% of these cancers initiate in the OSE [28]. Cell of origin of ovarian cancer has been debated and it is now believed that epithelial cells of the fallopian tube fimbria and ovary surface are the possible precursors of majority of ovarian cancers. Follicle depletion and stromal fibrosis along with increased inflammation are thought to provide a permissive environment (pre-metastatic niche) for ovarian cancer [29, 30].

Stromal fibrosis observed in the ovaries in the present study, is a characteristic feature of aging and is well documented in both mice and human aged ovaries. It leads to chronic inflammation and raises the risk of developing ovarian cancer by generating a microenvironment permissive for tumor growth [31, 32] similar to in other tissues [33, 34]. Metformin improves the immune landscape and abrogates age-associated fibrosis in both mice and human aged ovaries and as a result is helpful to reduce the risk of developing ovarian cancer [35]. Our results suggest that rather than the epithelial cells, it is the VSELs lodged in the OSE which possibly initiate ovarian cancer as discussed earlier [36]. This gets support from the published work [10] which also suggested that it is the small-sized, pluripotent stem cells that are greatly increased in numbers in ovarian cancer cases. These stem cells were observed in large numbers dispersed in the ovarian stroma in the present study and also in H&E stained smears from ascites fluid collected from an aged ovary. However, further characterization of these cells will be undertaken in future. Silvestris et al. [37] also discussed potential role of Ddx4^+^ ovarian stem cells in postmenopausal ovaries in carcinogenesis.

To conclude, aged mammalian ovaries similar to the adult ovaries harbor two populations of stem cells including VSELs and OSCs along with SCP-3 positive GCN. Differentiation of oocytes is blocked possibly due to a compromised stem cells niche. Hence, the oocytes in the nest are unable to assemble as primordial follicles. This is why transplanting MSCs helps improve ovarian function as they provide the paracrine support crucial for resident stem/progenitor cells to differentiate normally and overcome the meiotic-block. Resveratrol and Metformin also help improve the aged ovarian niche and thus facilitate differentiation of VSELs/OSCs into oocytes. VSELs are the cells of origin for ovarian cancers and further studies are warranted to decipher the stem cell biology in aged ovaries and ovarian cancer. Our study based on histology alone provides huge scope for further research. Detailed studies at the transcriptome level of the OSE cells need to be undertaken since intact ovary transcriptome data will not provide any information regarding the ovarian stem cells which comprise a very rare (less than 0.1% of total cells) population.

## Supplementary Information

**Additional file 1. **
