# Correction: Aged mice ovaries harbor stem cells and germ cell nests but fail to form follicles

**DOI:** 10.1186/s13048-023-01256-5

**Published:** 2023-08-14

**Authors:** Diksha Sharma, Deepa Bhartiya

**Affiliations:** Stem Cell Biology Department, ICMR-National Institute for Research in Reproductive Health, Jehangir Merwanji Street, 400 012 Mumbai, India

**Correction: J Ovarian Res 15:37 (2022)**

 10.1186/s13048-022-00968-4

Following publication of the original article [1], please note that Figs. 3A–G and 4 are composites prepared by putting together various fields as these cells are spread far apart on the slides.
