# Supplementary material for: Aged mice ovaries harbor stem cells and germ cell nests but fail to form follicles
Source: J Ovarian Res. 2022 Mar 23;15:37. doi: 10.1186/s13048-022-00968-4 (PMC8944102; doi:10.1186/s13048-022-00968-4)
Supplement: Supplementary file 1 — Additional file 1. [file 13048_2022_968_MOESM1_ESM.docx]

**Concern expressed by the reviewer and editor**

The conclusion that loss of primordial follicles results in senescence due to stem cell dysfunction is not supported by the data. The objective and results in the manuscript support that a stem cell population persists in aged ovaries. The results support this conclusion but **provide no evidence that they would function to form primordial follicles in young ovaries, but can't in adult ovaries, due to age-related niche changes.**

Our reply

The reviewer and the editor need to realize that this article on aged ovaries currently under review in JOR is an extension of our recently published article mentioned below.

[Diksha Sharma](https://pubmed.ncbi.nlm.nih.gov/?term=Sharma+D&cauthor_id=34455541), [Deepa Bhartiya](https://pubmed.ncbi.nlm.nih.gov/?term=Bhartiya+D&cauthor_id=34455541). **Stem cells in adult mice ovaries form germ cell nests, undergo meiosis, neo-oogenesis and follicle assembly on regular basis during estrus cycle**. Stem Cell Rev Rep. 2021 Oct;17(5):1695-1711. doi: 10.1007/s12015-021-10237-4.

This article is a major breakthrough in the field. We showed, without any genetic manipulation, how stem cells located in the ovary surface epithelium function across estrus cycle, how they form germ cell nests on regular basis which further differentiate into oocytes. Various events like GCN formation, Balbiani bodies, ring canals, meiosis and oocyte differentiation – which are well described in fetal/neonatal ovaries – also exist in adult ovaries. A distinct expression of SCP-3 was observed with nuclear expression and hence meiosis limited to estrus/metestrus (pl see Fig on next page)

Furthermore, FACS sorted, green fluorescent protein (GFP) positive VSELs upon transplantation resulted in GFP positive GCN suggesting crucial role for VSELs in adult ovaries. Results suggest that various events described during oogenesis and follicle assembly in fetal ovaries are recapitulated on regular basis in adult ovary and result in the formation of follicles. Moreover, GFP+ FACS sorted ovarian stem cells expressing SSEA-1 upon transplantation in wild type mice resulted in GFP+ germ cell nests in wild type adult ovaries. BrdU uptake was also studied in MVH positive stem cells that also differentiated into oocytes.

Distinct changes on OSE cell smears during different stages of estrus cycle. Epithelial cells remain pale stained and with abundant cytoplasm (K). Stem cells are darkly stained and spherical in shape, presence interspersed amongst the epithelial cells. Note presence of single and dividing doublets during proestrus. GCN were observed maximally during estrus and metestrus stage. Stem cell activity was minimal during diestrus. Scale: 20 μm A-D 10X, E–H 20X, I-L 40X. Figs M-P show SCP-3 expression in cells collected during different stages of estrus cycle. SCP-3 was cytoplasmic during diestrus and proestrus stages of estrus cycle and became nuclear (suggestive of meiosis) during estrus and metestrus stage. Note the changing chromatin pattern during different stages of estrus cycle showing progression of cells through prophase of meiosis 1. Scale bar is 5 μm

In fact, the work also attracted a commentary by Prof Kakar and Prof Ratajczak.

Dr Kakar is Editor-in-Chief of JOR.

[**Paper of October Issue of Stem Cell Reviews and Reports Presents a Novel View on Oogenesis in Adult Mammalian Ovaries.**](https://pubmed.ncbi.nlm.nih.gov/34448117/) Kakar SS, Ratajczak MZ. Stem Cell Rev Rep. 2021 Oct;17(5):1519-1520. doi: 10.1007/s12015-021-10248-1.

Once these results were obtained that ovarian stem cells are functional in adult ovaries and undergo neo-oogenesis in regular basis in adult ovaries, Diksha answered two questions – how stem cells biology gets altered in aged ovaries (currently under review in JOR) and how they get affected by neonatal exposure to endocrine disruption (under review).

***Thus, we have already published evidence that ovarian stem cells function to form primordial follicles in young ovaries, and in current article we show that these stem cells can't function normally in aged ovaries, due to age-related niche changes.***

We look forward to the decision of JOR. We have again carefully read the manuscript to avoid any ambiguity and made changes to further clarify and improved our language.
